# *NOTCH2* VUS Interpretation in Pediatric Cholestasis: A 365-Variant Landscape and Genotype–Phenotype Analysis of 7 Cases

**DOI:** 10.3390/genes17101259

**Published:** 2026-10-11

**Authors:** Brett J. Hoskins, Tiziano Pramparo, Shannon M. Vandriel, Chaowapong Jarasvaraparn, Michael J. Wilsey, Voytek Slowik, Lakshmi Kunam, Janis M. Stoll, Ruben E. Quiros-Tejeira, Simon Lam, Wikrom Karnsakul

**Affiliations:** 1 Division of Pediatric Gastroenterology, Hepatology and Nutrition, Department of Pediatrics, Riley Hospital for Children, Indiana University School of Medicine, Indianapolis, IN 46202, USA; cjarasva@iu.edu; 2 Mirum Pharmaceuticals, Inc., Foster City, CA 94404, USA; tpramparo@gmail.com (T.P.); shannon.vandriel@mirumpharma.com (S.M.V.); 3 Department of Gastroenterology, Johns Hopkins All Children’s Hospital, St. Petersburg, FL 33701, USA; mwilsey1@jhmi.edu; 4 Division of Pediatric Gastroenterology, Hepatology and Nutrition, Children’s Mercy—Kansas City, Kansas City, MO 64108, USA; vslowik@cmh.edu (V.S.); lakshmikunam98@gmail.com (L.K.); 5 Division of Pediatric Gastroenterology, Hepatology and Nutrition, Department of Pediatrics, Washington University School of Medicine, St. Louis, MO 63110, USA; janis.stoll@wustl.edu; 6 Division of Pediatric Gastroenterology, Hepatology and Nutrition, University of Nebraska Medical Center, Omaha, NE 68198, USA; rquiros@unmc.edu; 7 Department of Pediatrics, Cumming School of Medicine, University of Calgary, Calgary, AB T2N 4N1, Canada; simon.lam@albertahealthservices.ca; 8 Division of Pediatric Gastroenterology, Hepatology and Nutrition, Department of Pediatrics, The Johns Hopkins University School of Medicine, Baltimore, MD 21205, USA; wkarnsa1@jhmi.edu

**Keywords:** Alagille syndrome, cholestasis, domain mapping, genetic testing, precision medicine, variant interpretation, variant of uncertain significance

## Abstract

**Background/Objectives**: Interpretation of *NOTCH2* variants of uncertain significance (VUSs) remains challenging in pediatric cholestasis and suspected Alagille syndrome (ALGS) due to incomplete penetrance, phenotypic variability, and extensive genetic variation. Although disease-specific variant classification criteria have identified pathogenic hotspots within epidermal growth factor (EGF)-like repeats 8–12 and ankyrin repeat domains 3–6, characterizing these VUSs requires a robust framework. **Methods**: We reviewed aggregate deidentified data from 519 multigene cholestasis panel entries and evaluated genotype–phenotype data from a consortium cohort of 7 pediatric patients with heterozygous *NOTCH2* VUSs using a clinical–genomic prioritization framework informed by disease-specific ACMG criteria and domain location. **Results**: Across the 519 entries, 365 unique *NOTCH2* VUSs were identified, none of which overlapped with 69 previously reported ALGS-associated variants or localized to established hotspots, with over 60% occurring outside recognized functional domains. Among the 7 consortium cases, one patient carrying a cysteine-altering variant (p.Cys593Arg) in EGF-like domain 15 demonstrated a strong ALGS-compatible phenotype, while alternative diagnoses in other cases included trisomy 21 and congenital hepatic fibrosis with polycystic kidney disease. **Conclusions**: Among 365 unique *NOTCH2* VUSs identified through pediatric cholestasis panel testing, none overlapped with previously reported ALGS-associated variants or localized to established pathogenic hotspots. However, a cysteine-altering variant outside canonical hotspots occurred in a patient with a strong ALGS-compatible phenotype, highlighting the importance of integrating variant characteristics, domain location, detailed phenotyping, and alternative diagnoses when prioritizing *NOTCH2* VUSs for further evaluation.

## 1. Introduction

Genetic testing has become increasingly integrated into the evaluation of pediatric cholestasis, resulting in growing identification of variants of uncertain significance (VUSs) in genes associated with inherited cholestatic disorders [1,2]. Among these, *NOTCH2* presents a particular interpretive challenge because of extensive genetic variation, incomplete penetrance, and marked phenotypic heterogeneity [3,4]. *NOTCH2*-associated Alagille syndrome (ALGS) accounts for approximately 2–7% of ALGS cases and often demonstrates attenuated multisystem involvement compared with *JAG1*-mediated disease [4,5].

The NOTCH signaling pathway is essential for intrahepatic bile duct development. *NOTCH2* signaling regulates biliary epithelial cell specification and bile duct morphogenesis, and murine models have demonstrated that *NOTCH2* deficiency produces severe bile duct paucity whereas *Notch1* deletion does not [6,7]. Domain-level analyses have identified pathogenic enrichment within EGF-like repeats 8–12 and ANK repeats 3–6, particularly among cysteine-altering variants and splice-disrupting alleles [4,8]. However, many *NOTCH2* variants identified during broad gene-panel testing do not localize to these regions, complicating assessment of their potential clinical relevance [9].

Recently, the Global ALAgille Alliance (GALA) study group proposed disease-specific American College of Medical Genetics and Genomics (ACMG) refinements for *NOTCH2* variant interpretation and demonstrated substantial reclassification of previously reported pathogenic variants to VUSs [4]. Together with recently published ACMG recommendations emphasizing phenotype-driven reporting of VUSs, these findings highlight the need for interpretation frameworks that integrate variant-level evidence with clinical phenotype while avoiding overinterpretation of uncertain findings [4,10].

We therefore evaluated the spectrum of *NOTCH2* VUSs identified through pediatric cholestasis gene-panel testing and applied a clinical–genomic prioritization framework to 7 pediatric cases with heterozygous *NOTCH2* VUSs contributed by participating consortium centers.

## 2. Materials and Methods

We conducted a multicenter retrospective study within an ongoing pediatric cholestasis consortium established to harmonize phenotype definitions, genotype interpretation, and clinical data abstraction across participating pediatric hepatology centers in North America. Participating centers were identified through collaborative pediatric hepatology networks with expertise in cholestatic liver disease and genomic testing.

The study incorporated two complementary datasets with distinct analytical purposes. First, aggregate de-identified data from a sponsored multigene cholestasis panel program comprising 519 testing entries with *NOTCH2* analysis, previously described by Hoskins et al. [1], were reviewed to characterize the overall landscape of *NOTCH2* VUSs. The 519 entries represent testing entries rather than necessarily unique individuals, whereas the 365 VUSs identified within these entries represent unique *NOTCH2* variants after deduplication. Variants were compared against the 69 ALGS-associated *NOTCH2* variants reported by Vandriel et al. (2025) [4]. Domain mapping was performed to assess localization within established pathogenic hotspots, including EGF-like repeats 8–12 and ANK repeats 3–6. Analyses of this aggregate dataset were descriptive.

Second, participating consortium centers contributed detailed genotype–phenotype data from 7 pediatric patients harboring heterozygous *NOTCH2* VUSs for focused clinical–genomic correlation. These 7 patients represented all eligible cases contributed by participating centers during the study period. Because the aggregate panel dataset was de-identified, potential overlap between the 7 consortium cases and the 519 testing entries could not be determined. All genetic testing had been performed clinically prior to inclusion in this study using next-generation sequencing assays at CLIA-certified laboratories. Variant classifications reflected the original clinical laboratory interpretations at the time of testing. Variant nomenclature was standardized to the NM_024408.4 MANE Select reference transcript for reporting in this study. Additional heterozygous cholestasis-associated genetic findings identified on prior clinical testing were recorded when present.

Clinical features, including cholestasis phenotype, bile duct paucity, characteristic facies, vertebral anomalies, renal and vascular involvement, alternative etiologies, and additional genetic findings, were reviewed alongside variant type and domain location. Published disease-specific *NOTCH2* ACMG criteria were considered as part of variant interpretation [4]; however, the original clinical laboratory VUSs classification at the time of testing was retained, and the study did not independently reclassify variants.

Phenotype strength and priority for further evaluation were assigned using a descriptive, expert-informed framework rather than a validated quantitative scoring system. Phenotype strength was categorized as weak, partial, or strong based on the number and specificity of ALGS-compatible hepatic and extrahepatic features and the presence of competing diagnoses. Priority for further evaluation was categorized as low, moderate, or high and integrated phenotype strength with variant characteristics, including variant type, domain location, potential structural relevance such as cysteine alteration, and alternative diagnoses. Variants with limited phenotype compatibility, less compelling variant characteristics, or a strong alternative explanation were generally assigned lower priority, whereas variants combining greater phenotype compatibility with potentially relevant variant characteristics were assigned higher priority. These categories were intended to support clinical–genomic prioritization and did not constitute pathogenicity classifications.

This study was approved by the Institutional Review Board at Johns Hopkins University School of Medicine (approval number IRB00299237) with additional approval obtained at all participating centers. Given the retrospective design and use of de-identified data, waiver of informed consent was granted. The study was conducted in accordance with the principles of the Declaration of Helsinki and the Declaration of Istanbul and was reported in accordance with the Strengthening the Reporting of Observational Studies in Epidemiology (STROBE) guidelines.

## 3. Results

### 3.1. Landscape of NOTCH2 VUSs

Among 519 total testing entries, 365 unique *NOTCH2* VUSs were identified. None overlapped with the 69 ALGS-associated variants previously reported by Vandriel et al. (2025) [4]. No variants localized within the established ALGS pathogenic hotspots of EGF-like repeats 8–12 or ANK repeats 3–6. Only 28 variants (7.7%) localized within the extended EGF cluster spanning repeats 13–23, whereas greater than 60% (*n* = 223) occurred outside recognized functional domains. Approximately 40 variants localized to the terminal exon 34 tail region, where truncating *NOTCH2* variants have been associated with Hajdu–Cheney syndrome [11]. Eight variants involved noncanonical splice regions, while no canonical splice-site variants were identified. Only one cysteine-altering variant, p.Cys593Arg, was observed. Additional heterozygous cholestasis-associated VUSs, none of which are independently diagnostic of a defined cholestatic disorder, were also present in multiple cases across the consortium datasets. Overall, the aggregate dataset demonstrated a marked lack of overlap between the identified *NOTCH2* VUSs and currently recognized features of ALGS-associated *NOTCH2* variation. The landscape and domain distribution of *NOTCH2* VUSs in the aggregate panel dataset are summarized in Table 1.

### 3.2. Clinical–Genomic Correlation of 7 Cases

Clinical and genomic characteristics of the 7 consortium cases are summarized in Table 2, with detailed clinical, laboratory, imaging, and histopathologic findings provided in Supplementary Table S1. Among the 7 consortium cases, 5 variants were missense and 2 involved noncanonical splice-region alterations. Additional heterozygous cholestasis-associated VUSs were present in multiple cases, including variants involving *CFTR*, *PKHD1*, *NPC1*, *POLG*, *ALDOB*, and *CC2D2A*. Four patients demonstrated partial or strong ALGS-compatible phenotypes (Patients 1, 3, 6, and 7). Literature review did not identify prior reports of these 7 exact variants in ALGS or cholestatic liver disease, and none overlapped with previously reported ALGS-associated *NOTCH2* variants. Clinical prioritization therefore incorporated variant type, domain location, phenotype compatibility, and the presence of alternative diagnoses. These priority assignments were intended to guide further evaluation and were not equivalent to ACMG pathogenicity classifications. Patients 4 and 6 carried noncanonical splice-region variants (c.2027-4A>G and c.2479+3G>C, respectively); in the absence of RNA studies, an effect on splicing could not be established for either variant.

Patient 7 carried the cysteine-altering p.Cys593Arg variant within EGF-like domain 15. Although outside the canonical EGF 8–12 hotspot, the cysteine-altering nature of the variant, together with severe cholestasis, markedly elevated serum bile acids (total >400 µmol/L), severe pruritus, liver fibrosis, ALGS-compatible facies, and ischemic stroke, resulted in the highest priority for further evaluation. Ileal bile acid transporter inhibitor therapy using maralixibat (Livmarli; Mirum Pharmaceuticals, Inc., Foster City, CA, USA) was initiated based on the patient’s clinical phenotype despite the absence of formal variant reclassification and was associated with sustained improvement in cholestatic pruritus and serum bile acid levels over 2 years of follow-up. This therapeutic response was considered a clinical observation and was not used as evidence supporting pathogenicity of p.Cys593Arg.

Patient 1 demonstrated neonatal cholestasis, ALGS-compatible facies, renal anomaly, and vascular involvement in the setting of p.Arg2256Cys, a missense variant in the C-terminal tail region outside recognized hotspots. The patient underwent Kasai portoenterostomy at 2 months of age for possible biliary atresia and subsequently experienced progressive disease resulting in listing for liver transplantation and death. Because biliary atresia was not definitively confirmed and multiple ALGS-compatible features were present, an underlying syndromic cholestatic disorder could not be excluded; however, the competing diagnosis limited attribution of the phenotype to the *NOTCH2* VUSs. The phenotype was therefore considered partially compatible with ALGS and assigned moderate priority for further evaluation. Patient 6 carried the noncanonical +3 splice-region variant c.2479+3G>C near EGF-like domains 22–23 and presented with neonatal cholestasis, ALGS-compatible facies, and butterfly vertebrae, resulting in moderate priority for further evaluation.

Several additional cases illustrated the importance of alternative etiologies and phenotype correlation. Patient 3 demonstrated bile duct paucity and neonatal cholestasis but also had trisomy 21, lowering the prior probability that the distal *NOTCH2* VUS represented the primary disease driver. Patient 5 carried a variant near EGF-like domain 13 but also had clinical findings consistent with congenital hepatic fibrosis with polycystic kidney disease, providing a compelling alternative explanation for the phenotype. Patients 2, 4, and 5 demonstrated weak ALGS-compatible phenotypes and were assigned low interpretive priority.

## 4. Discussion

This multicenter consortium study demonstrates a striking divergence between the broad landscape of *NOTCH2* VUSs identified through pediatric cholestasis gene-panel testing and currently recognized features of ALGS-associated *NOTCH2* variation. Among 365 unique VUSs, none overlapped with the 69 previously reported ALGS-associated variants or localized within the canonical EGF-like 8–12 or ANK 3–6 pathogenic hotspots [4,8]. At the same time, detailed genotype–phenotype evaluation of 7 pediatric cases identified a cysteine-altering variant outside these canonical hotspots in a patient with a strong ALGS-compatible phenotype, illustrating why domain location alone cannot determine the clinical relevance of an individual VUS.

The observed distribution demonstrates that most *NOTCH2* VUSs identified in this diagnostic dataset lack currently recognized features of ALGS-associated *NOTCH2* variation. However, individual-level phenotype, segregation, population-frequency, and functional data were not available for the 365-VUS dataset, precluding conclusions regarding the pathogenicity or benignity of individual variants. Population reference datasets, including Genome Aggregation Database (gnomAD) and All of Us, have expanded characterization of rare variation across ancestries and provide important evidence for variant interpretation [12,13]. The GALA study demonstrated that additional genome sequencing detected 4 previously unrecognized pathogenic variants in patients with clinically suspected ALGS, highlighting that expanded sequencing approaches can improve diagnostic yield in carefully selected phenotype-positive, genotype-negative patients [4]. This interpretation is also consistent with recently published ACMG recommendations emphasizing phenotype-driven reporting of VUSs, in which variants identified in symptomatic patients undergoing diagnostic evaluation remain clinically relevant, whereas incidental VUSs identified through asymptomatic testing are generally not recommended for reporting [10].

These data also illustrate both the utility and limitations of domain-level interpretation. Prior studies demonstrated enrichment of pathogenic variants within EGF-like repeats 8–12 and ANK repeats 3–6, regions critical for ligand binding and downstream transcriptional activation [3,8]. Domain location may therefore contribute useful evidence when prioritizing a VUS but should not be used as an exclusion criterion for pathogenicity. Nonetheless, Patient 7 highlights this limitation, as cysteine-altering variants outside canonical hotspots may still warrant heightened concern when accompanied by a compelling phenotype. This interpretation is further complicated by the recognized phenotypic divergence between *JAG1*- and *NOTCH2*-associated ALGS. *NOTCH2*-associated disease may present with attenuated or atypical clinical manifestations, and the absence of a classic ALGS presentation alone should not exclude pathogenicity when variants occur within relevant domains or exhibit features suggestive of structural disruption [4]. Disease-specific ACMG frameworks developed by the GALA study group similarly prioritize cysteine-disrupting EGF variants because of their potential structural consequences [4].

The study also highlights the importance of integrating competing clinical diagnoses into interpretation. Possible biliary atresia, trisomy 21, congenital hepatic fibrosis with polycystic kidney disease, idiopathic neonatal hepatitis, and self-limited cholestasis all provided alternative or competing explanations for the observed phenotypes in individual cases. Their presence reduced confidence in attributing the clinical phenotype to a *NOTCH2* VUS but did not establish that the *NOTCH2* variant itself was benign. Current guidelines emphasize that diagnosis and management of pediatric genetic cholestatic disease should integrate clinical, laboratory, and genetic data rather than rely on VUS status alone [14]. This phenotype-anchored approach is also supported by a recent companion multicenter study from the same collaborative cohort evaluating monoallelic PFIC-associated VUSs in children [15].

The clinical implications also extend beyond variant interpretation. Emerging evidence supports the efficacy of ileal bile acid transporter inhibitors including maralixibat (Livmarli) and odevixibat (Bylvay; Ipsen, Paris, France) for cholestatic pruritus in clinically compatible ALGS regardless of molecular confirmation [16,17]. Patient 7 demonstrated severe cholestatic disease with markedly elevated bile acids, severe pruritus, and strong ALGS-compatible features despite the lack of formal variant reclassification. Phenotype-driven initiation of maralixibat was associated with sustained improvement in cholestatic pruritus and serum bile acid levels over 2 years of follow-up. While this single-patient therapeutic response does not establish a causal relationship between the *NOTCH2* variant and treatment response or provide evidence of variant pathogenicity, it supports the use of the overall clinical phenotype to guide therapeutic decisions while variant interpretation remains unresolved.

This study has several limitations. The retrospective design and small number of deeply phenotyped consortium cases limit generalizability, and case ascertainment may introduce selection bias. Individual-level phenotype, segregation, population-frequency, and functional data were not available for the 365 unique VUSs in the aggregate dataset, limiting conclusions regarding the pathogenicity or benignity of individual variants. Because the aggregate data consisted of testing entries rather than confirmed unique individuals, repeated testing of the same individual could not be excluded. The aggregate dataset also represents patients undergoing diagnostic cholestasis testing rather than a general-population reference cohort. Clinical and genetic data were heterogeneous across participating centers, segregation data were limited, and no functional validation was performed for the 7 variants. Variant classifications reflected the original clinical laboratory interpretations at the time of testing and were not independently reclassified in this study. Finally, phenotype strength and priority for further evaluation were descriptive, expert-informed assessments rather than validated scoring systems. These limitations emphasize that the proposed framework is intended to prioritize VUSs for further evaluation rather than assign pathogenicity.

## 5. Conclusions

Among 365 unique *NOTCH2* VUSs identified through pediatric cholestasis panel testing, none overlapped with previously reported ALGS-associated variants or localized to established pathogenic hotspots. However, a cysteine-altering variant outside canonical hotspots occurred in a patient with a strong ALGS-compatible phenotype, demonstrating that domain location alone cannot determine the clinical relevance of an individual VUS. Integrating variant characteristics, domain location, detailed phenotyping, and alternative diagnoses provides a practical framework for prioritizing *NOTCH2* VUSs for further evaluation while avoiding overinterpretation of uncertain findings.

## Figures and Tables

**Table 1:** *NOTCH2* variants of uncertain significance identified through multigene cholestasis panel testing.

Category	Findings
Total *NOTCH2* test entries	519
Unique *NOTCH2* VUSs	365
Overlap with known ALGS *NOTCH2* variants [4]	0/69 variants (none matched)
Variants in ALGS hotspots (EGF 8–12 or ANK 3–6)	0
Variants in extended EGF cluster (EGF 13–23)	28 (7.7%)
Variants outside all functional domains	>60% (*n* = 223)
Noncanonical splice-region variants (+3, −3 to −5)	8
Canonical splice-site variants (±1/±2)	0
Variants in terminal exon 34 (tail region)	~40
Cysteine-altering variants	1 cysteine-altering variant (p.Cys593Arg)
Overall interpretation	Marked lack of overlap with currently recognized ALGS-associated *NOTCH2* variants and pathogenic hotspots.

Abbreviations: ALGS, Alagille syndrome; ANK, ankyrin repeat; EGF, epidermal growth factor; VUSs, variants of uncertain significance.

**Table 2:** Combined clinical and genomic summary of 7 cases with *NOTCH2* variants of uncertain significance.

Patient	*NOTCH2* Variant (Protein)	Domain/Region	Hotspot Relevance	Key ALGS-Compatible Features	Additional Cholestasis-Associated VUSs	Alternative Diagnosis	Phenotype Strength	Priority for Further Evaluation
1	NM_024408.4:c.6766C>T (p.Arg2256Cys)	Tail region	Outside hotspot	Neonatal cholestasis; facies; renal anomaly; vascular involvement	*CC2D2A*, *NPC1*, *POLG*	Possible biliary atresia	Partial	Moderate
2	NM_024408.4:c.6893G>A (p.Arg2298Gln)	Tail region	Outside hotspot	None	*CFTR*, *PKHD1*	—	Weak	Low
3	NM_024408.4:c.7377G>A (p.Met2459Ile)	Tail region	Outside hotspot	Neonatal cholestasis; bile duct paucity	*POLG*	Trisomy 21	Partial	Low–Moderate
4	NM_024408.4:c.2027-4A>G (splice-region)	EGF-like 13	Near but outside hotspot	Neonatal cholestasis	None	Idiopathic neonatal hepatitis	Weak	Low
5	NM_024408.4:c.1804G>A (p.Ala602Thr)	EGF-like 13	Near but outside hotspot	Cholestasis; renal anomaly; congenital hepatic fibrosis	*CFTR*	Congenital hepatic fibrosis with polycystic kidney disease	Weak	Low
6	NM_024408.4:c.2479+3G>C (splice-region)	EGF-like 22–23	Outside hotspot	Neonatal cholestasis; facies; butterfly vertebrae	*ALDOB*, *NPC1*	—	Partial	Moderate
7	NM_024408.4:c.1777T>C (p.Cys593Arg)	EGF-like 15	Outside hotspot; cysteine-altering variant	Severe cholestasis; high bile acids; severe pruritus; facies; ischemic stroke; liver fibrosis	None	—	Strong	High (highest priority)

Abbreviations: ACMG, American College of Medical Genetics and Genomics; ALGS, Alagille syndrome; EGF, epidermal growth factor; VUS, variant of uncertain significance. All *NOTCH2* variants are reported using the NM_024408.4 MANE Select reference transcript. Phenotype strength and priority for further evaluation represent descriptive, expert-informed assessments integrating ALGS-compatible clinical features, variant characteristics and domain location, and alternative diagnoses; they are not validated scores or ACMG pathogenicity classifications.

## Data Availability

The data that support the findings of this study are available from the corresponding author upon reasonable request. Individual-level genotype–phenotype data are not publicly available due to privacy and ethical restrictions related to the inclusion of patients with rare genetic variants.

## Supplementary Materials

The following supporting information can be downloaded at: https://www.mdpi.com/article/10.3390/genes17101259/s1, Table S1: Detailed clinical, laboratory, imaging, and histopathologic characteristics of seven pediatric patients with *NOTCH2* variants of uncertain significance.

## Abbreviations

ACMG	American College of Medical Genetics and Genomics
ADEK	Fat-soluble vitamin (A, D, E, and K) supplementation
ALGS	Alagille syndrome
ALP	Alkaline phosphatase
ALT	Alanine aminotransferase
ANK	Ankyrin repeat
AST	Aspartate aminotransferase
CLIA	Clinical Laboratory Improvement Amendments
CT	Computed tomography
DBili	Direct bilirubin
DDW	Digestive Disease Week
DNA	Deoxyribonucleic acid
EGF	Epidermal growth factor
GALA	Global ALAgille Alliance
GGT	Gamma-glutamyl transferase
gnomAD	Genome Aggregation Database
HSM	Hepatosplenomegaly
IRB	Institutional Review Board
MRI	Magnetic resonance imaging
TBili	Total bilirubin
US	Ultrasound
VUS	Variant of uncertain significance
