# Supplementary material for: NOTCH2 VUS Interpretation in Pediatric Cholestasis: A 365-Variant Landscape and Genotype–Phenotype Analysis of 7 Cases
Source: Genes (Basel). 2026 Oct 11;17(10):1259. doi: 10.3390/genes17101259 (PMC13704217; doi:10.3390/genes17101259)
Supplement: Supplementary file 1 [file genes-17-01259-s001.zip › genes-4598140-supplementary.pdf]

Supplementary Table S1. Detailed clinical, laboratory, imaging, and histopathologic characteristics of seven pediatric patients with *NOTCH2* variants of uncertain significance.

Patient	Age at Onset	Age at Testing	Sex	Ethnicity	Jaundice	HSM	*NOTCH2* Features	Extrahepatic Features	Peak AST	Peak ALT	Peak ALP	Peak TBili	Peak DBili	Peak GGT	Imaging Performed	Medications	Liver Biopsy, Findings	Liver Transplantation (or other surgery)	Deceased	Alternative Diagnosis
**1**	1 to <2 months	2 to <3 months	Female	African American	Yes	Yes	Liver involvement, neonatal cholestasis, characteristic facies, renal anomaly, vascular involvement	Encephalopathy, developmental regression, seizures, renal anomaly, pulmonary involvement	66	149	405	4.8	4.0	259	US: Absent gallbladder	ADEK, ursodiol	No	Listed<br><br>Kasai portoenterostomy at 2 months of age	Yes	Possible biliary atresia versus syndromic cholestatic liver diseases
**2**	1 year	2 to 5 years	Male	Unknown	Yes	No	None	None	221	278	380	4.4	3.3	518	US: Normal	None	No	No	No	—
**3**	<1 month	1 to <2 months	Female	Caucasian	Yes	No	Liver involvement, neonatal cholestasis	Developmental regression, seizures	94	64	777	5.2	3.7	135	US: Irregular gallbladder	None	Yes, paucity of bile ducts	No	No	Trisomy 21
**4**	2 to <3 months	6 to <12 months	Female	Caucasian	Yes	No	Liver involvement, neonatal cholestasis	None	100	63	437	2.7	0.6	52	US: Normal	None	No	No	No	Idiopathic neonatal hepatitis
**5**	2 to 5 years	6 to 10 years	Female	Caucasian	No	Yes	Liver involvement, renal anomaly (polycystic kidney disease)	Polycystic kidney disease	42	22	146	0.2	0.1	15	US: HSM<br><br>CT/MRI: Focal nodular hyperplasia, HSM	None	No	No	No	Congenital hepatic fibrosis
**6**	3 to <4 months	1 year	Male	African American	No	No	Liver involvement, neonatal cholestasis, characteristic facies, butterfly vertebrae	Butterfly vertebrae	81	86	244	0.2	0.1	34	US: Normal	Famotidine	No	No	Yes	—
**7**	1 to <2 months	3 to <4 months	Male	Caucasian	Yes	Yes	Liver involvement, neonatal cholestasis, characteristic facies, ischemic stroke	Cerebral palsy, ischemic stroke	213	363	522	4.7	3.1	706	US: Diffuse hepatic parenchymal heterogeneity<br><br>MRI Brain:	ADEK, maralixibat, ursodiol	No	No	No	—

Abbreviations: ADEK, fat-soluble vitamin (A, D, E, and K) supplementation; AST, aspartate aminotransferase; ALP, alkaline phosphatase; ALT, alanine aminotransferase; CT, computed tomography; DBili, direct bilirubin; GGT, gamma-glutamyl transferase; HSM, hepatosplenomegaly; MRI, magnetic resonance imaging; TBili, total bilirubin; US, ultrasound.
